# Depletion of polycomb repressive complex 2 core component EED impairs fetal hematopoiesis

**DOI:** 10.1038/cddis.2017.163

**Published:** 2017-04-13

**Authors:** Wenhua Yu, Fang Zhang, Shiyan Wang, Yi Fu, Jiahuan Chen, Xiaodong Liang, Huangying Le, William T Pu, Bing Zhang

**Affiliations:** 1 Key Laboratory of Systems Biomedicine, Shanghai Center for Systems Biomedicine, Shanghai Jiao Tong University, Shanghai 200240, China; 2 Department of Cardiology, Boston Children's Hospital, Boston, MA 02115, USA; 3 Harvard Stem Cell Institute, Cambridge, MA 02138, USA

## Abstract

Polycomb repressive complex 2 (PRC2), a H3K27me3 methyltransferase complex, promotes the development of many organs by silencing ectopic transcription program. However, currently little is known about the role of PRC2 in blood and vascular development. In this study, we interrogated the function of embryonic ectoderm development (EED), a core PRC2 component, in both endothelial and hematopoietic tissues by inactivating a floxed murine EED allele with Tie2Cre, which catalyzes recombination in endothelial and hematopoietic lineages. Murine EED^fl/fl^;Tie2Cre (EED^CKO^) embryos died at embryonic day (E) 13.5. We did not observe structural abnormalities of blood vessels or cardiac valves, suggesting that EED is dispensable in endothelial cells for initial steps of vascular development. EED^CKO^ embryos were pale and had abnormal livers. Flow cytometry of fetal liver cells showed that EED depletion significantly impeded erythroid maturation. There was a corresponding increase in myeloid progenitors and granulocytes and macrophages, suggesting an attenuated differentiation path in myeloid lineages. Moreover, EED depletion impaired the generation of hematopoietic stem cells. Collectively, our study demonstrates that within Tie2Cre-recombined embryonic cells, EED is required for proper erythropoiesis and for formation of hematopoietic progenitor and stem cells, but is dispensable for endothelial lineage commitment and early vascular patterning.

Polycomb repressive complex 2 (PRC2) represses gene transcription by trimethylating histone H3 on lysine 27 (H3K27me3). The core PRC2 complex is comprised of embryonic ectoderm development (EED), Suppressor of Zeste12 homolog (SUZ12), retinoblastoma-associated protein 48 (RbAp48), and a catalytic subunit, either Enhancer of Zeste Homolog 1 or 2 (EZH1 or EZH2).^1, 2^ EZH1 and EZH2 are mutually exclusive in the same PRC2 complex and are partially redundant at certain developmental and disease stages.^3, 4^ EED is a core component of PRC2 complex that interacts with EZH1 or EZH2 through a WD40 domain. In addition, EED recognizes H3K27me3 through an aromatic cage structure to recruit PRC2 to established H3K27me3 and thereby enforce maintenance of this repressive epigenetic mark.^5, 6^

PRC2 has essential roles of regulating cell fate commitment, organogenesis, homeostasis and disease-related tissue remodeling.^1^ In early developing mouse embryos, homozygous *Eed* null mutation abolished global H3K27 methylation and caused defects in primitive streak formation and fetal lethality at E9.5.^7, 8^ EZH2 also controls the fate of multiple types of tissue progenitors.^9^ Depletion of EZH2 in epidermal precursor cells promoted premature epidermal differentiation and barrier acquisition.^10, 11^ EZH2 also had developmental-stage-specific roles in regulating cardiomyocyte differentiation and proliferation.^12, 13^ In adult vasculature, EZH2 was required for VEGF-induced silencing of vasohibin1 (VASH1), which promoted angiogenesis in ovarian cancer.^14^

Both endothelial cell (EC) and hematopoietic cells originate from a common progenitor, the hemangioblast. Hemogenic ECs within the yolk sac give rise to the first population of hematopoietic stem cells (HSCs), which migrate to aorta-gonad mesonephros (AGM) and then to the fetal liver, which then becomes the main site of fetal hematopoiesis. Fetal liver HSCs establish the common myeloid progenitor (CMP) and the common lymphoid progenitor (CLP), multipotent progenitors that differentiate into the major blood lineages. CMPs further differentiate into megakaryocyte–erythrocyte progenitors (MEPs), precursors of erythrocytes and megakaryocytes, and granulocyte–macrophage progenitors (GMPs), precursors of granulocytes and monocyte/macrophages.^15, 16, 17^

Functional links between PRC2, hematopoiesis, and hematopoietic malignancies have been recently unveiled. Human genetic studies revealed that mutations affecting *EZH2*, *EED*, *SUZ12*, and *JARID2,* encoding PRC2 subunits and associated proteins, cause hematopoietic diseases including myelodysplastic syndrome, T-cell acute lymphoblastic leukemia, and B cell lymphoma.^18, 19, 20, 21^ However, the functions of PRC2 in hematopoietic development are currently less clear. Conditional deletion of *Ezh2* in hematopoietic and vascular lineages of mouse embryos by Tie2Cre was lethal at mid-gestation due to insufficient expansion of hematopoietic stem/progenitor cells (HSCs).^22^ In contrast, conditional deletion of EED in HSCs by Vav^Cre^ did not significantly influence fetal HSC hemostasis but became significant in the adulthood.^23^ Thus, the role of PRC2 and its component subunits in blood and vessel development require elucidation.

In this study, we used Tie2Cre to inactivate EED in developing blood and vascular lineages. Because there is no functional redundant ortholog of EED, this should abolish PRC2 function in these lineages and circumvent functional redundancy between EZH1 and EZH2. We found that EED is dispensable for EC specification and initial development into vessels and cardiac valve precursors, but is essential for normal population of the fetal liver by HSCs and for erythropoiesis. Combined with previous studies on PRC2, our results illustrated a tissue-context-dependent and developmental-stage-specific role of PRC2 in controlling hematopoiesis.

## Results

### EED is required in the Tie2Cre lineage for normal embryonic development

EED^flox^ mice were described previously and develop normally.^23^ Although heterozygosity for a null *Eed* allele was previously reported to cause myelo- and lympohoproliferative defects,^24^ Tie2Cre;EED^fl/wt^ mice survived normally to adulthood and were fertile (Table 1). However, no live Tie2Cre;EED^fl/fl^ (EED conditional knockout, abbreviated as EED^CKO^) mice were observed at birth (Table 1), indicating that EED is required in the Tie2Cre lineage for normal embryonic development.

**Figure 1:**
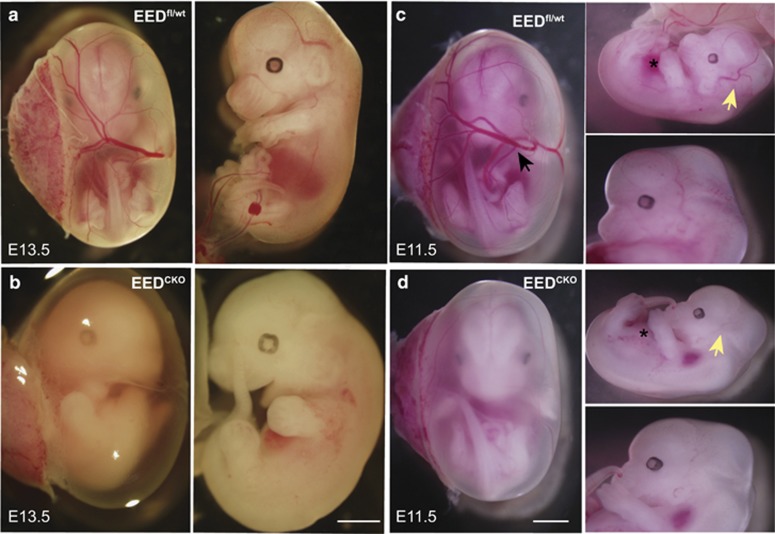
EED^CKO^ mouse is embryonic lethal at mid-gestation. (**a** and **b**) Images of wild-type and EED^CKO^ embryos at E13.5. Most EED^CKO^ embryos had died by this stage. (**c** and **d**) Images of wild-type and EED^CKO^ embryos at E11.5. EED^CKO^ embryos show significant paleness and absence of blood-perfused vasculature. Asterisks overlie the fetal liver. Black arrow points to a vitelline vessel. Yellow arrow indicates primary head vein. Bar, 0.2 cm (**b**) or 0.3 cm (**d**)

To determine the time of embryonic lethality, we analyzed litters at E11.5 and E13.5. At E11.5, EED^CKO^ were present at the expected Mendelian frequency, whereas 70% of mutant embryos were no longer viable at E13.5 (Table 1, Figures 1a and b). However, E11.5 EED^CKO^ embryos already showed developmental abnormalities, as they lacked large blood-perfused vessels including vitelline veins in the yolk sac (Figures 1c and d, black arrow) and primary head veins of the embryo (Figures 1c and d, yellow arrow). The region occupied by the liver was also notably pale (Figures 1c and d, asterisk).

### EED deletion did not significantly alter the vascular patterning

**Figure 2:**
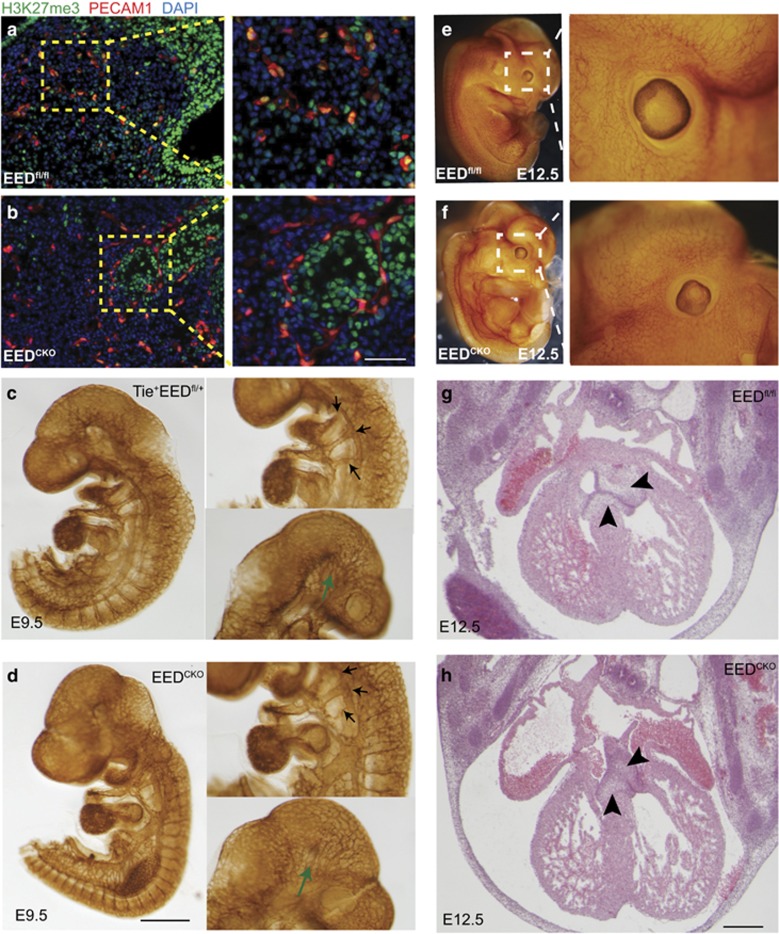
Normal blood vessel and cardiac valve development in EED^CKO^. (**a** and **b**) Immunofluorescent images of skin tissue from E12.5 stage embryos. Overall, H3K27me3 signal was reduced in endothelial cells. Bar, 0.1 cm. (**c** and **d**) PECAM1 whole-mount staining of E9.5 embryos. Black arrows point at branchial arch arteries. Green arrows point at cerebral vascular plexus. Bar, 0.1 cm. (**e** and **f**) PECAM1 whole-mount staining of E12.5 embryos. (**g** and **h**) H&E staining of E12.5 embryonic heart section. Arrowheads point at atrioventricular endocardial cushion. Bar, 50 *μ*m

Given that EED is an essential component of PRC2, which deposits H3K27me3, we performed immunostaining to measure bulk H3K27me3 levels. Immunofluorescent staining demonstrated substantially weaker H3K27me3 signal in endothelial cells (Figures 2a and b), consistent with efficient EED deletion in these cells by Tie2Cre. This also suggested the possibility that the observed absence of blood-filled vessels in EED^CKO^ embryos was due to abnormal vascular development (Figures 2a and b).

To test this possibility, we examined blood vessels of wild-type and EED^CKO^ embryos at E9.5 and E12.5 by PECAM1 whole-mount staining, a marker of vascular endothelial cell. Unexpectedly, at E9.5, there was no global angiodysplasia observed in EED mutants. The large vessels such as the pharyngeal arch arteries, midline dorsa aorta, cardinal vein, intersomitic vessels, and large cerebral vessels were well developed in EED^CKO^ (Figures 2c and d). The small vessels formed complete vascular plexuses with similar density and pattern to control littermates. We also checked the vasculature of E12.5 embryos and found no significant difference in vascular density or pattern in EED^CKO^ compared with control (Figures 2e and f).

Endothelial cells of the heart contribute to heart valve mesenchyme formation through endothelial-to-mesenchymal transition, and Tie2Cre-driven mutations in *EC* genes have caused lethal heart valve defects.^25, 26^ To test whether the deletion of EED results in valve hypoplasia, we examined heart valves in E12.5 EED^CKO^ and control littermates. Cardiac valve size and morphology were comparable between mutant and wild-type embryos (Figures 2g and h). Taken together, these data suggest that EED depletion did not significantly influence vessel or valve formation, and therefore are unlikely to account for the observed embryonic lethality.

### Deletion of EED attenuated the myeloid lineages commitment

**Figure 3:**
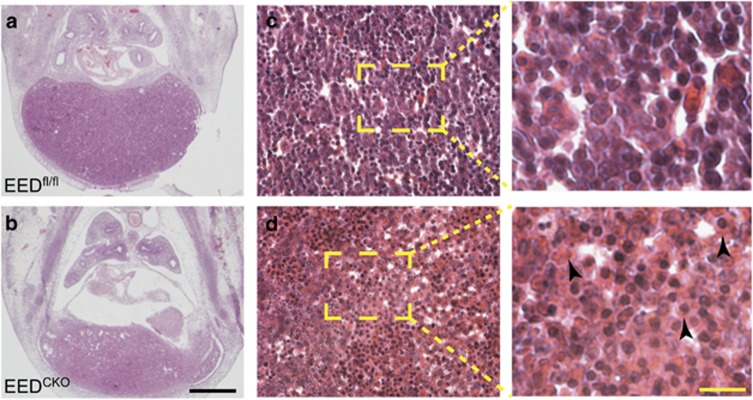
Hepatic abnormalities of EED^CKO^ embryos. (**a** and **b**) H&E staining of E12.5 histological section through the liver. Arrowheads indicate swollen cells with eosinophilic cytoplasm. Black bar, 50 *μ*m. Yellow bar, 10 *μ*m

Tie2Cre drives recombination in almost all hematopoietic cells, in addition to endothelial cells.^27^ As the deletion of PRC2 has been reported to induce defective hematopoiesis,^23, 24^ we hypothesized that death of EED^CKO^ embryos is due to the abnormal hematopoiesis. Between E11 and E15.5, the fetal liver is the primary site of hematopoiesis.^15, 28^ Given the abnormal appearance of the fetal liver on gross embryo inspection (Figures 1c and d), we examined fetal liver morphology on H&E-stained histological sections and found that EED^CKO^ liver size was significantly reduced (Figures 3a and b). Hepatic structure was poorly organized, and the cytoplasm of blood cells within the liver were swollen and more eosinophilic than controls (Figure 3b). All together, these data suggested disrupted fetal hematopoiesis.

**Figure 4:**
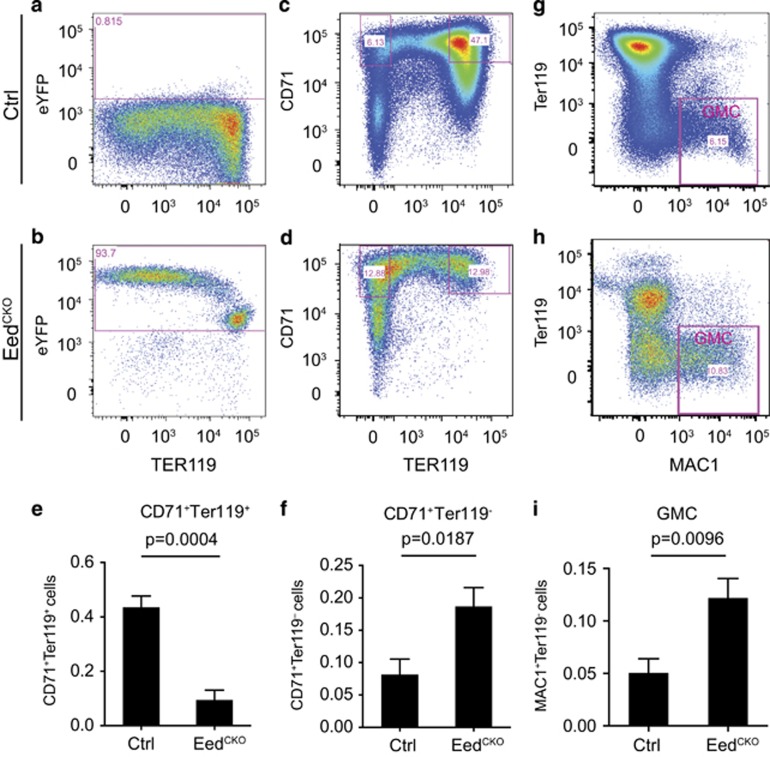
EED^CKO^ caused defective erythrocyte maturation. Flow cytometry analysis of hematopoietic cells from hepatic liver. (**a** and **b**) Detection of hematopoietic cells that expressed YFP from Tie2Cre-recombined Rosa26^fsYFP^. (**c** and **d**) Flow cytometry analysis of erythroblasts (CD71^+^Ter119^+^). (**e**) Quantitative analysis of C71^+^Ter119^+^ erythroblasts. Unpaired *t*-test, *n*=4–7. (**f** and **g**) Flow cytometry analysis of less mature erythroblasts (CD71^+^Ter119^−^). (**h**) Quantitative analysis of CD71^+^Ter119^–^ erythroblasts. Unpaired *t*-test, *n*=4–7. (**i** and **j**) Flow cytometry analysis of the granulocyte lineage (MAC1^+^Ter119^–^). (**k**) Quantitative analysis of granulocyte lineage. Unpaired *t*-test, *n*=4–7

To check the status of hematopoiesis, especially erythropoiesis, whose deficiency could have contributed to the pale appearance of EED^CKO^ embryos, we isolated hematopoietic cells from fetal liver and examined their lineage composition by flow cytometry. Using a Cre-activated Rosa26-floxed-stop-YFP (Rosa26^fsYFP^) reporter, we determined that over 90% of hematopoietic cells were YFP^+^ (Figures 4a and b), confirming efficient Tie2Cre-mediated recombination within the hematopoietic lineage.^27^ We then evaluated erythropoiesis using flow cytometry to measure lineage differentiation markers. CD71^+^Ter119^+^ cells, representing more mature erythroblasts, were dramatically depleted compared with control (9.4% *versus* 43%, *P*=0.0004; Figures 4c–e). In contrast, less mature CD71^+^Ter119^−^ erythroblasts were significantly increased (18.6% *versus* 8.1%, *P*=0.0001, Figures 4c, d and f), which suggested that EED is required for erythroblast maturation.

Erythroblasts arise from a common myeloid progenitor (CMP), which also differentiates into the granulocyte–macrophage progenitor (GMP), which subsequently differentiates into granulocytes and macrophages (GMC).^17, 29^ The granulocytes and macrophages express the cell surface marker Mac1 and lack Ter119.^17^ By flow cytometry, MAC1^+^Ter119^–^ cells increased almost 2.4-fold (*P*=0.0001) in EED^CKO^ (Figures 4g–i). This result suggests that EED depletion biases CMP differentiation towards GMC at the expense of the myeloid lineage.

### EED depletion exhausted the HSC pool

**Figure 5:**
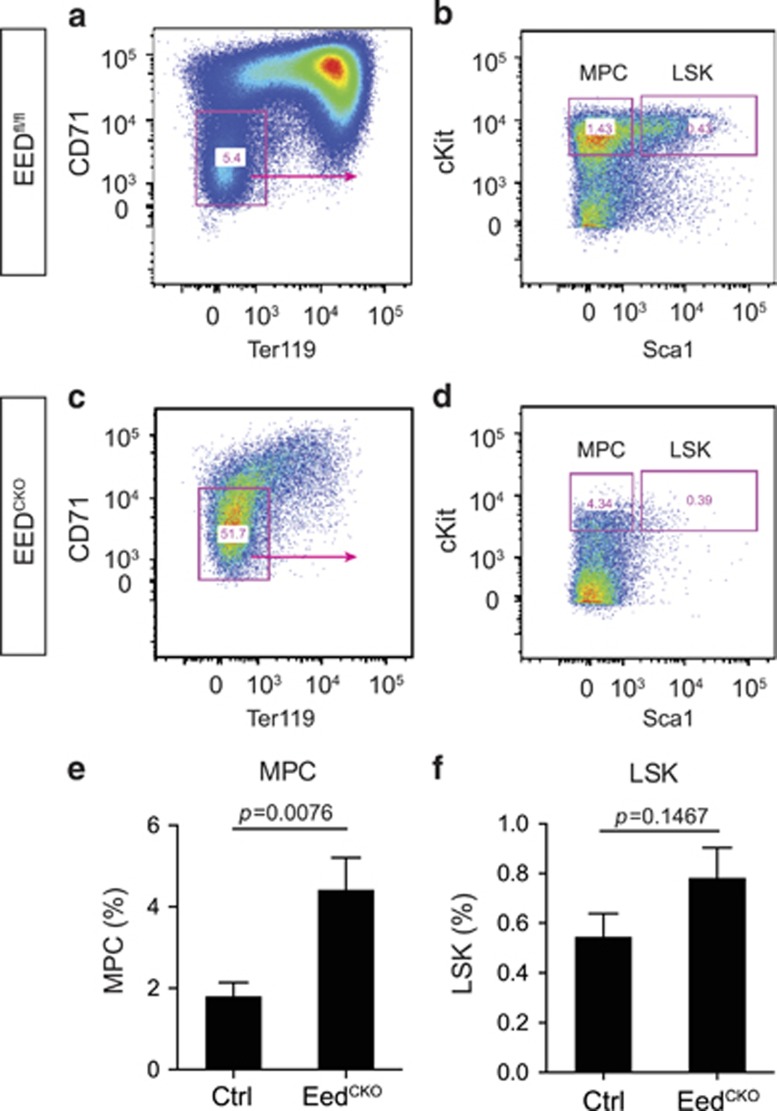
*Eed* deletion resulted in defective development of myeloid progenitor cells. (**a**–**d**) Flow cytometry analysis of myeloid progenitor cells (MPCs) and LSK cells from control and EED^CKO^ fetal liver. (**e**) Quantitative analysis of MPCs. Unpaired *t*-test, *n*=3–6. (**f**) Quantitative analysis of LSK cells. Unpaired *t*-test, *n*=3–6

Erythroblasts and granulocytes are derived from myeloid progenitors. To investigate whether EED is required for normal formation of myeloid progenitor cells, we measured multipotent hematopoietic and myeloid progenitors by flow cytometry. Myeloid progenitor cell (MPC) lacks canonical erythrocyte maker of CD71 and Ter119. Thus, we first isolated the CD71^–^Ter119^–^ blood cells and further stained them with typical progenitor markers of Sca1 and cKit to gate the MPC (Figures 5a–d). The percentage of CD71^–^Ter119^–^cKit^+^Sca1^–^ myeloid progenitors was increased by 2.5-fold in EED^CKO^ compared with control littermates (*P*=0.0076; Figure 5e), indicating EED depletion hindered MPC differentiation or promotes its proliferation or self-renewal. The total number of CD71^–^Ter119^–^ Sca1^+^cKit^+^ (LSK) cells was not altered in EED^CKO^ although they were significantly depleted in C71^−^Ter119^−^ population (*P*=0.1467; Figure 5f).

**Figure 6:**
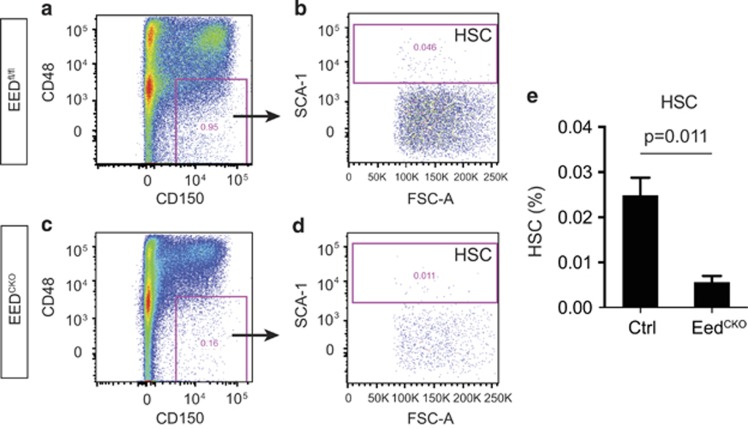
*Eed* deletion resulted in defective HSC development. (**a**–**d**) FACS strategy defining HSC cells in wild-type and EED^CKO^ mouse. (**e**) Quantitative analysis of HSC cells. Unpaired *t*-test, *n*=3–5

The depletion of cKit^+^ cells in CD71^−^Ter119^−^ population indicates the loss of HSC in EED^CKO^ mouse. HSCs are hematopoiesis-committed multipotent cells that self-renew and give rise to all hematopoietic lineages. To test whether PRC2 regulates HSC generation or homeostasis, we stained for cell surface markers C48 and C150. Within Lin^−^CD48^–^CD150^+^ cells, HSC cells are the subset that expresses the pan-stem cell marker Sca1^23^ (Figures 6a–d). Consistent with a previous study showing the impaired expansion of HSC cells following EZH2 depletion, the HSC population in EED^KO^ was significantly reduced (0.025 *versus* 0.0056%, *P*=0.011; Figure 6e). Taken together, these data indicate that EED deletion impaired the generation or maintenance of HSCs.

## Discussion

The PRC2 complex is a master regulator of differentiation programs and organogenesis. In this study, we depleted EED, a core component of PRC2. Unlike EZH1 or EZH2, there is no known functional redundancy for EED within PRC2, and therefore EED inactivation should delineate required PRC2 functions. Inactivation of EED^flox^ using Tie2Cre illustrated that EED depletion did not strongly affect vascular development but significantly disrupted erythroid maturation as well as HSC formation, homeostasis, and differentiation, which together led to embryonic lethality. These findings reinforced current knowledge of the essential role of PRC2 in regulating cell differentiation and homeostasis, while unveiling new roles for PRC2 in regulating hematopoiesis.

### PRC2 and hematopoiesis

PRC2 has been recently shown to regulate the development and malignancy of multiple hematopoietic lineages,^20, 22, 23, 30, 31^ but its roles in these processes are still imprecisely defined. Ectopic expression of EZH2 in bone marrow HSCs promoted HSC proliferation and myeloproliferative disease.^32^ Conversely, inactivating mutations of PRC2 core members SUZ12 and EED, induced by *N*-ethyl-*N*-nitrosourea (ENU), disrupted HSC regeneration.^30^ Depletion of EZH2 by Tie2Cre caused defective HSC development in the fetal liver, but did not compromise adult HSC capacity to reconstitute the bone marrow.^22^ However, a recent study reported that deletion of EED by Vav^Cre^, which is active in hematopoietic cells, was compatible with fetal survival, leading to the opposite conclusion that PRC2 is dispensable for fetal HSC differentiation and maintenance.^23^ Results from these EZH2 and EED knockout studies have been difficult to compare due to the different Cre drivers, functional redundancy of EZH1/2, and non-canonical roles of EZH2 that are not dependent on EED.^33^ Furthermore, a recent study reported that EED functions as a scaffold protein that interacts with catalytic components of the PRC1 complex,^34^ adding further complexity to comparisons between EZH2 and EED knockout studies.

Our study reconciles the results of the Tie2Cre;Ezh2^fl/fl^ and Vav^Cre^;EED^fl/fl^ studies to a large extent by deleting EED^fl/fl^ with Tie2Cre. We found that EED depletion by Tie2Cre disrupted HSC homeostasis and severely impaired erythropoiesis. We reason the different results from two experiments might be due to the earlier onset of Tie2Cre deletion in hemangioblasts. EED-deficient hemangioblasts might be defective in maitenance or further differentiation, whereas this defect may not be exposed when EED ablation occurs in HSCs or their descendants. Although lethal effects of EED depletion on hematopoiesis may require its inactivation in angioblasts, the functional defect may not occur in angioblasts, as we have previously observed that altering PRC2 in progenitors may not have functional consequences until a later developmental time point,^12^ perhaps reflecting a 'memory' effect of the chromatin landscape.

Tie2Cre also efficiently targets endothelial cells, which contribute to form a stem cell niche to nurse multiple types of hemotapoietic progenitors including HSC by paracrine mechanisms.^15, 35^ Hence, even though we did not observe vascular abnormalities in the EED^CKO^ mutants, it is possible that Tie2Cre-mediated EED deletion in EC could influence this paracrine mechanism and thereby cell non-autonomously influence HSC maintenance and differentiation. To test this hypothesis, a more EC-restricted Cre such as CDH5^CreERT2^^3^ will need to be used.^36^

### PRC2 and vascular development

Recently Tie2Cre;EZH2^fl/fl^ mice were reported to have defective vascular integrity and severe hemorrhage evident at E12.5 and due to the activation of *Mmp9*.^37^ In our study, we did not find significant hemorrhage up to E12.5, a day before death. Thus, it is less likely that embryonic lethality of our Tie2Cre;EED^fl/fl^ mutants was due to vascular leakage. The divergent results might result from technical differences in mouse strain background or kinetics of Cre inactivation. An alternative intriguing possibility is that the more severe vascular phenotype of EZH2^fl/fl^ mutants reflects a non-canonical, EED-independent role of EZH2 in regulating endothelial cell genes such as *Mmp9*. Another potential contributor to the divergent results is the recently reported role of EED in PRC1 activity.^34^

In adult mice, siRNA-mediated *Ezh2* knockdown was used to show that EZH2 promotes tumor angiogenesis by repressing anti-angiogenic factor *Vash1*.^14^ We did not find an essential role for EED in developmental angiogenesis. These studies may point out differences in the dependence of developmental *versus* tumor angiogenesis on PRC2 or they may further highlight non-canonical roles of EZH2. Alternatively, technical differences in the method of gene inactivation may have led to divergent conclusions. A genetic deletion of *Eed* or *Ezh2* by inducible CDH5^CreETR2^ in the adult mouse would help to solidify our current understanding of the role of PRC2 in angiogenesis and even possibly generate new insights into the functions of EED and EZH2 within PRC2 and in non-canonical roles.^33^

### PRC2 and hematopoietic malignancies

Mutations of genes encoding PRC2 subunits have been linked to multiple types of human hematopoietic malignancies. Somatic mutation of *EED* or chromosome deletion of *EZH2* led to myelodysplastic syndrome and related neoplasm, which is consistent to our and others studies that depletion of EZH2 and EED in hematopoietic cells impaired erythroblast generation, erythrocyte maturation, and caused cytopenia.^31, 32, 38^ Moreover, structural rearrange of *EZH2*, *SUZ12*, and *EED* both in mouse and human results in the T-cell acute lymphoblast leukemia.^20^ The malignant transformation in hematopoietic cells lacking PRC2 suggests that compensating for PRC2 deficiency may be a productive therapeutic strategy for some hematopoietic disorders. Consistent with this idea, overexpression of EZH2 in mice augmented HSC regeneration and prevented the HSC exhaustion during the transplantation assays.^32^ However, elevation of EZH2 and other PRC2 components is associated with aggressive forms in solid cancers and related to cancer progression.^39, 40^ As a result, PRC2 inhibition has emerged as a potential strategy cancer treatment strategy, and is currently being tested in clinical trials. Given the deleterious effects of PRC2 inhibition on HSCs and hematopoiesis illuminated in this study and others, the side effects and therapeutic index of these agents needs to be carefully evaluated.

## Materials and Methods

### Mice

All animal procedures were approved by Institutional Animal Care and Use Committee (IACUC) of Shanghai Jiao Tong University and Boston Children's Hospital. The EED^flox^, Tie2Cre, and Rosa26fsYFP alleles were described previously.^23, 41, 42^ All mouse strains were maintained in a C57BL/6 and 129 mixed background. EED^fl/fl^ female mice were mated with Tie2^Cre^;EED^fl/wt^ male mice to generate Tie^Cre^;EED^fl/fl^ embyros, to avoid germline deletion that can result from Tie2Cre transmitted through the female germline. Gestational age of embryos was determined by checking vaginal plugs, with noon of the day of the plug defined as embryonic day (E) E0.5.

### Immunofluorescent staining

Immunofluorescent staining of embryo sections was performed as described previously.^43, 44^ Cryopreserved embryos were sectioned, fixed in 4% PFA for 15 min, and blocked and permeabilized with Blocking buffer (1% BSA in PBS containing 5% goat serum) containing 0.1% Triton X-100. Following 1 h of blocking at room temperature, the sections were incubated with primary antibodies (H3K27me3, 17-622, Millipore; PECAM1, clone MEC13.3, BD Bioscience, San Diego, USA) overnight at 4 ˚C. The samples were washed three times with PBS containing 0.05% Tween-20, incubated with Alexa-conjugated secondary antibodies (1:200, Fisher Scientific, Shanghai, China) for 1 h and then counterstained with DAPI (1 *μ*g/ml, Roche) for 5 min before mounting. The images were captured on a Olympus FluoView FV1000 confocal microscope (China).

### Whole-mount staining

Whole-mount immunohistochemical staining of E9.5 and E12.5 mouse embryos using PECAM1 antibody was performed as reported previously.^43, 44^ Briefly, embryos were dissected from the uterus, fixed in 4% paraformaldehyde overnight, and dehydrated in 100% methanol until use. The embryo was quenched with 5% hydrogen peroxide in methanol to remove the endogenous peroxidase and then rehydrated in 75, 50, and 25% methanol before use. The blocking process was performed with incubation of blocking buffer (2% nonfat milk, 5% goat serum and 0.2% Triton X-100) for 2 h at room temperature. After blocking, PECAM1 antibody (MEC13.3, 1:200; BD Pharmingen) in blocking buffer was applied to embryos with gentle shaking overnight. The embryos were washed with PBS five times, incubated overnight with biotinylated goal anti-rat antibody (1:50, BD Pharmingen) in PBS containing 0.2% Triton X-100, and then incubated with HRP-conjugated streptavidin for 2 h before color development in DAB substrates (Vector Labs, Burlingame, CA, USA). The images were obtained using an SMZ800 stereo microscope.

### Flow cytometry

Fetal livers were isolated from E12.5 mouse embryos, triturated, and passed through 70 *μ*m nylon mesh to obtain a single-cell suspension. The RBCs were lysed in 1 × erythroid lysis buffer. The isolated cells were first incubated on ice with Fc-Block. For selection of Lin^−^ cells, the cells were incubated with biotin-conjugated antibodies against lineage-specific markers (Ter119[TER119] CD71; B220[RA3-6B2] Gr1[RB6-8C5]), and biotin was subsequently bound by PerCP-Cy5.5 conjugated streptavidin. The cells were stained as indicated for surface markers, including FITC-CD34, phycoerythrin(PE)-Cy7-conjugated Sca1(D7), allophycocyanin (APC)-Cy7-conjugated cKit (2B8), and PE-conjugated CD48 (HM48-1). These antibodies were purchased from BD Biosciences (San Jose, CA, USA), Thermo Fisher (Shanghai, China) and BioLegend (San Diego, CA, USA). The dead cells were excluded by staining with 7-AAD (1 *μ*g/ml, Thermo Fisher). Antibody-labeled cells were run on an LSR II/LSR Fortessa for analysis or on a FACSAria II for cell sorting. Flowjo software (Ashland, OR, USA) was used to analyze the cytometry data.

### Statistics

The results are shown as mean±S.E.M. Student's *t*-test was used to determine whether groups were significantly different.

## Figures and Tables

**Table 1:** Survival rate of Tie2^Cre^;EED^fl/fl^ knockout mouse

**Age**	**EED^fl/wt^**	**EED^fl/fl^**	**TieCre^+^EED^fl/wt^**	**TieCre^+^EED^fl/fl^**
P0	16 (33%)	16 (33%)	16 (33%)	0 (0%)
E13.5	16 (24%)	20 (29%)	18 (26%)	14 (4 viable=6%)
E11.5	30 (30%)	27 (27%)	20 (20%)	23 (23%)

Table indicates number and (%) of embryos found at each stage.
